# Acute Generalized Exanthematous Pustulosis Induced by Cetuximab

**DOI:** 10.7759/cureus.17309

**Published:** 2021-08-19

**Authors:** Sadia Masood, Mohammad Rizwan, Saira Fatima, Palwasha Jalil

**Affiliations:** 1 Dermatology, Aga Khan University Hospital, Karachi, PAK; 2 Histopathology, Aga Khan University, Karachi, PAK

**Keywords:** squamous cell carcinoma, acute generalized exanthematous pustulosis, cetuximab

## Abstract

A wide variety of diseases and drugs can cause cutaneous pustular eruptions. Acute generalized exanthematous pustulosis (AGEP) is a unique drug-induced dermatosis clinically presented as erythematous papular and pustular eruption, usually caused by certain systemic drugs. We are presenting a very rare association of AGEP with a biological agent, cetuximab. A male aged 66 years, who was recently diagnosed with a case of squamous cell carcinoma of glottis, presented in the dermatology clinic with a recent onset of fever and widespread pustular eruption over the face, trunk, and limbs. The eruption was noted after the injection of cetuximab given for his squamous cell carcinoma. The clinical history, typical physical findings, and histopathological features confirm the diagnosis of AGEP. The injection cetuximab was stopped and the patient was treated with some topical and systemic medications and the symptoms resolved completely in a few weeks. Our case is an interesting clinical presentation of AGEP due to cetuximab therapy and confirms that this is an extremely rare and proven adverse effect of cetuximab. To our knowledge, this is the first-ever reported case of AGEP associated with cetuximab. Physicians need to be aware of this unique but important side effect of cetuximab and perform a proper physical examination and specific investigations that can be useful to reach a final diagnosis.

## Introduction

Cutaneous adverse reactions to various systemically administered drugs are common. These reactions show various clinical features and mechanisms, but reports of pustular drug eruptions are infrequent. Acute generalized exanthematous pustulosis (AGEP) is a specific drug-induced dermatosis presented as fever with acute episodes of non-follicular sterile tiny pustules, which usually regress a few days after discontinuation of the drug [1]. The histopathologic features of lesions are very characteristic and reveal non-follicular subcorneal pustules associated with vasculitis changes. Treatment modalities for AGEP include withdrawal of offending agents, antipyretics, and systemic steroids [2]. It usually heals within days or sometimes in a few weeks. It can occur at any age, though it commonly affects middle-aged adults with a slight female predominance [3]. We are presenting a case of AGEP provoked by the administration of cetuximab in a patient with squamous cell carcinoma of the head and neck, though a variety of drugs have been implicated in this condition and the current literature highlighted that there are no reported cases of AGEP caused by cetuximab. 

## Case presentation

**Figure 1:**
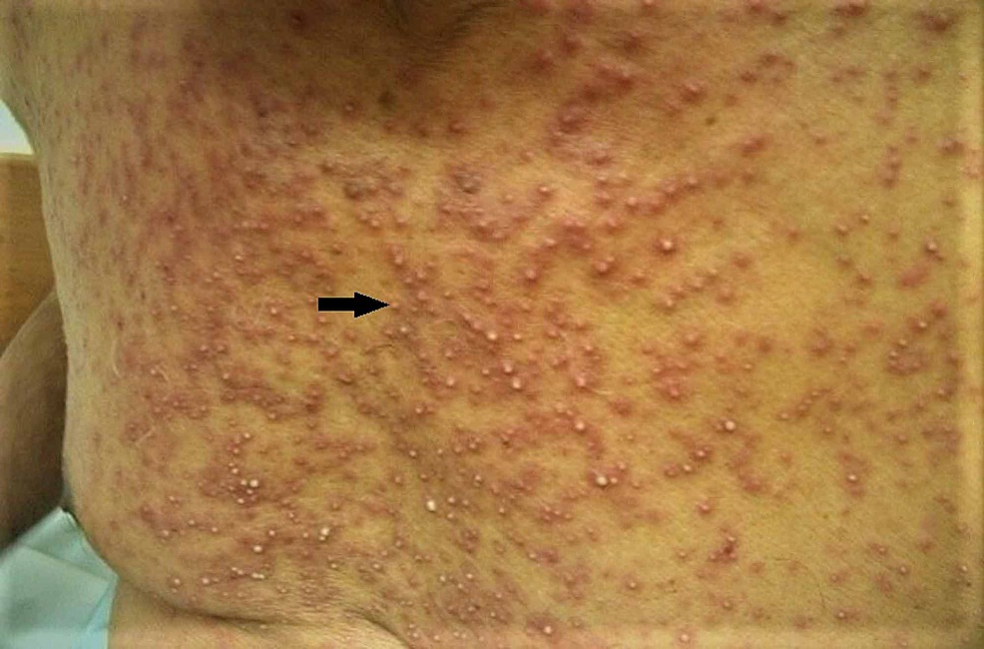
Multiple pinpoint pustules appeared on diffuse edematous and erythematous plaques and patches on trunk

**Figure 2:**
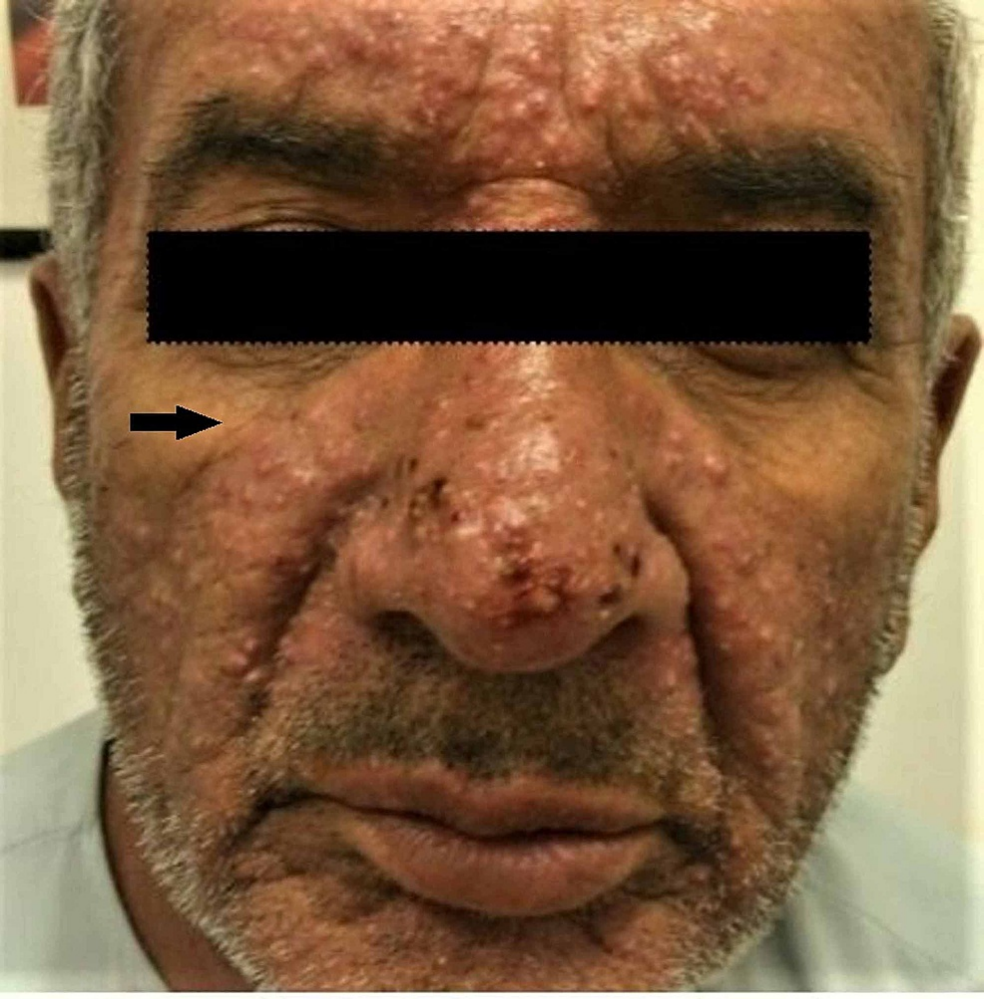
Multiple pinpoint pustules appeared on diffuse edematous and erythematous plaques and patches on face

A 66-year-old man, a known case of squamous cell carcinoma of the glottis, presented in the dermatology outpatient clinic with a complaint of fever of 39.6°C and sudden widespread non-pruritic, non-tender skin eruptions that had started a few days ago after the loading dose of cetuximab given for his squamous cell carcinoma (T3N0M0). Initially, the cutaneous rash was limited to the face but it rapidly spread to involve the trunk and limbs. The next day after the skin eruption, he developed a fever and sore throat. There was no family or personal history of any skin disorder. The dermatological examination revealed multiple pinpoint pustules that appeared on diffuse edematous and erythematous plaques and patches (Figures 1, 2).

**Figure 3:**
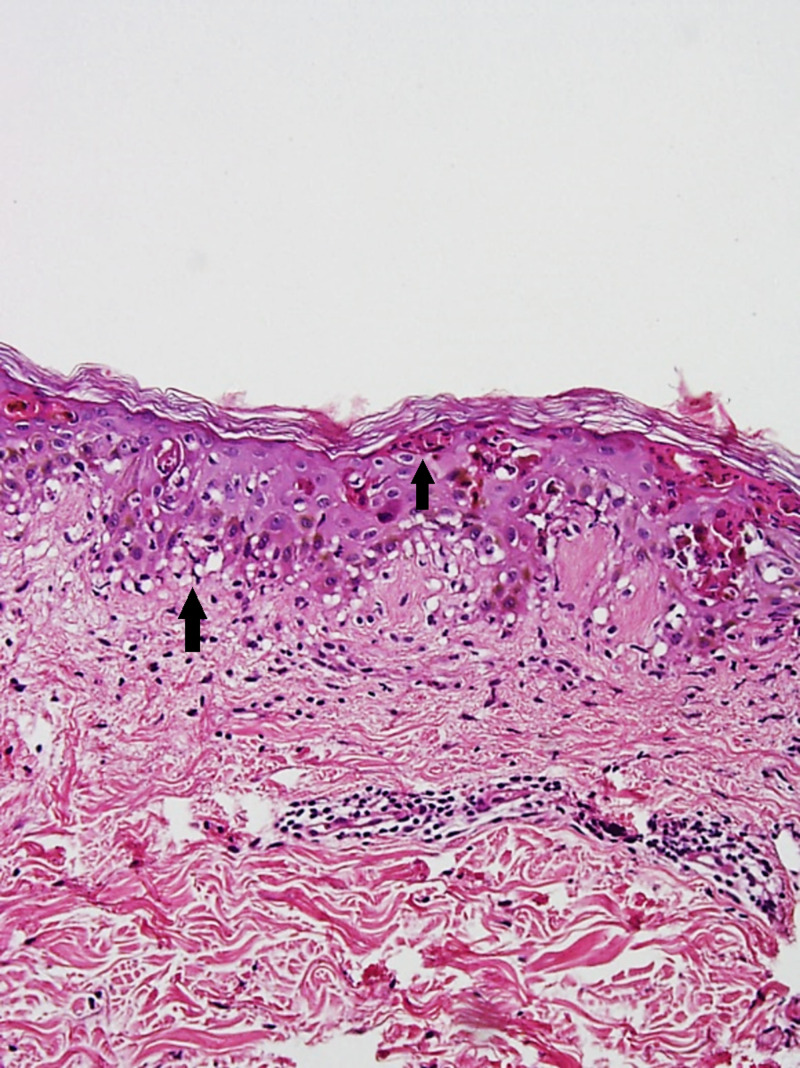
Marked basal damage, necrotic keratinocytes along with neutrophils below corneal layer

**Figure 4:**
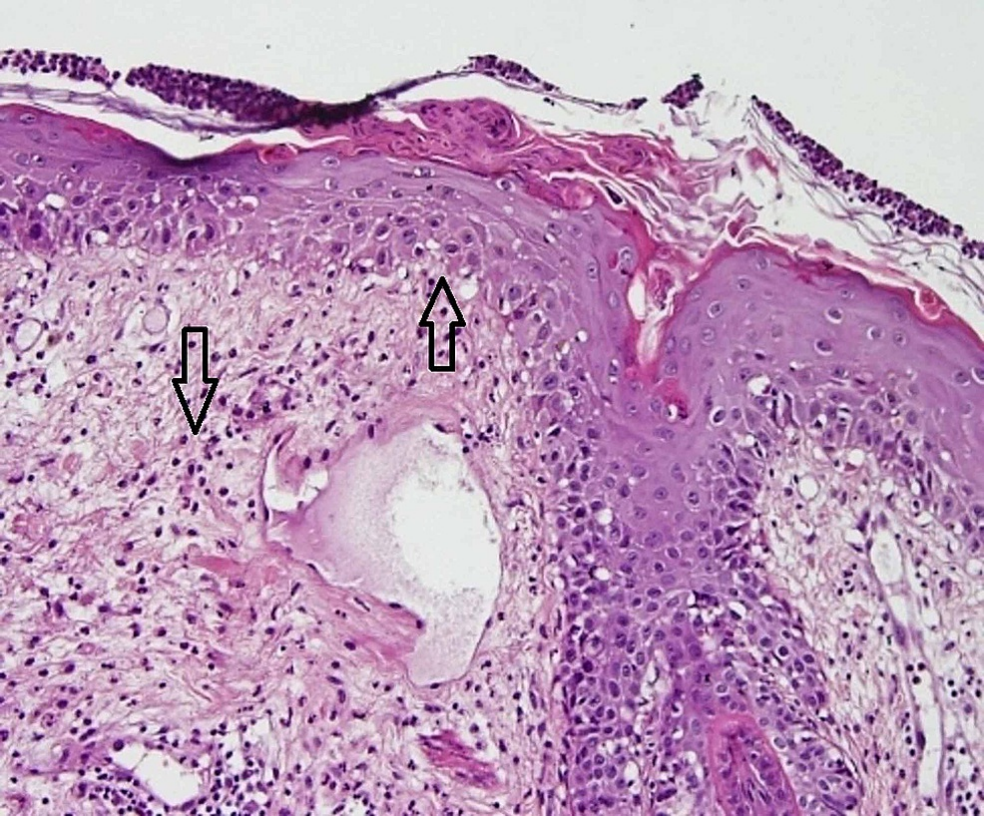
Subcorneal pustule formation with interface damage and mixed dermal infiltrate

These patches and plaques were predominantly present on the face, trunk, and bilateral limbs. Baseline investigations were normal except for mild eosinophilia (6.2%). Liver and renal function tests were normal. Bacterial cultures of pustules, peripheral blood, and urine were negative for any growth. Histopathologic examination of skin biopsy with hematoxylin-eosin stain displayed the presence of subcorneal pustule formation with marked basal damage, necrotic keratinocytes, and neutrophils below the corneal layer (Figures 3, 4).

Gram stains, special stains, and periodic acid Schiff were negative for fungi and bacteria. The clinical diagnosis of AGEP was made based on clinical history, physical examination, and investigations. The suspected culprit drug was withdrawn instantly and empirical antibiotics and systemic corticosteroids were timely instituted. Follow-up review after three weeks revealed complete resolution of skin lesions with mild post-inflammatory hyperpigmentation.

The patient became afebrile and the skin eruptions stopped progressing from the sixth day of the eruption. The patient was advised to regularly apply moisturizers. Another follow-up that was done after eight weeks showed complete improvement of skin lesions.

## Discussion

AGEP is a rapidly progressive erythroderma with tiny pustules on erythematous skin typically occurring within 24-48 hours of exposure to the offending drug and resolving rapidly with its cessation [4,5]. Beylot et al. initially earlier in 1980 introduced the term AGEP in French literature. He described specific pustular eruptions that erupt acutely after drug administration or a bout of infection in patients without a history of psoriasis [6]. The eruption is clinically manifesting as widespread follicular and non-follicular pustules on an erythematous background, which erupts quickly after using a culprit drug. In the cases of AGEP, the symptoms of skin are always associated with fever [7,8]. The histopathology of skin lesions demonstrates spongiform intraepidermal and subcorneal pustules with marked papillary dermal edema, perivascular neutrophilic infiltrate, and eosinophilic exocytosis [9]. Treatment modalities for AGEP include withdrawal of offending agents, antipyretics, and systemic steroids [10]. It usually heals within days or sometimes in a few weeks. Elderly and immunocompromised patients with extensive skin eruption may sometimes need to be hospitalized for electrolyte and fluid replacement [7]. The pathologic mechanism of AGEP has not been extensively studied. It is a T-cell-mediated inflammatory condition that involves cytotoxic CD8, CD4 T cells, and inflammatory cytokines and chemokines. CD4+ drug-specific T cells produce a huge number of granulocyte-macrophage colony-stimulating factors while interleukin 8 is involved in the accumulation of neutrophils in the tissues. In AGEP, the T-helper 17 cells are involved in the activation, recruitment, and migration of neutrophils [8].

AGEP has been described after administration of several drugs, including macrolides, amoxicillin, dexamethasone, paracetamol, nadoxolol, carbamazepine, vancomycin, and nifedipine [9]. Biological agents, compared to conventional chemotherapy regimens, have lower toxicity profiles. Although, some specific adverse effects like allergic reactions and skin rashes may limit their therapeutic use. The current case report highlights an interesting clinical association of AGEP after the use of cetuximab, which was administered for squamous cell carcinoma of the glottis [10]. Cetuximab is a chimeric monoclonal antibody that effectively binds the epidermal growth factor receptor (EGFR). It is an FDA-approved drug for locally invasive squamous cell carcinoma of the head and neck with concomitant radiotherapy and refractory metastatic colorectal cancer [11]. Busam et al. first described cutaneous adverse effects of EGFR, since then several reports have been published. The spectrum of cutaneous reactions to the EGFR inhibitors include follicular rashes 60-80%, paronychia 6-12%, dry skin 4-35%, hypersensitivity reactions 2-3%, hair changes, and mucositis 2-36%. Other common side effects include photosensitivity, hypomagnesemia, and less commonly pulmonary and cardiac toxicity [12]. However, up to 10% of patients developed severe reactions. There are no case studies in the literature showing the relationship between cetuximab and AGEP. We are well aware that our article is the first description of a cetuximab-related pustuloderma.

Cetuximab, like other biologics, is the potential treatment option for patients who are intolerant to chemotherapy as it targets the specific molecular pathways that affect the cancer evolution. But clinicians should be alert for its possible severe skin toxicity like AGEP. Close surveillance may be required for early treatment interruption as well as to prevent further cutaneous and systemic complications [13]. We believe that the patient is the first case of cetuximab-induced AGEP. Clinicians should be aware of this unique but important side effect and perform a proper physical examination and specific investigations that can be useful in recognizing the implicated drug and reaching a final diagnosis.

## Conclusions

This is a unique and a rare case of AGEP related to cetuximab ever reported in the literature. These skin reactions are very unusual, and we have intended to draw attention to these drug reactions related to biological agents in the etiology of such pustular skin rashes. It would help the physicians to keep AGEP in mind while administering biological agents for cancer therapy.
